# Improvement of Pest Resistance in Transgenic Tobacco Plants Expressing dsRNA of an Insect-Associated Gene *EcR*

**DOI:** 10.1371/journal.pone.0038572

**Published:** 2012-06-07

**Authors:** Jin-Qi Zhu, Shumin Liu, Yao Ma, Jia-Qi Zhang, Hai-Sheng Qi, Zhao-Jun Wei, Qiong Yao, Wen-Qing Zhang, Sheng Li

**Affiliations:** 1 Key Laboratory of Insect Developmental and Evolutionary Biology, Institute of Plant Physiology and Ecology, Shanghai Institutes for Biological Sciences, Chinese Academy of Sciences, Shanghai, China; 2 School of Biotechnology and Food Engineering, Hefei University of Technology, Hefei, China; 3 State Key Laboratory of Biocontrol and Institute of Entomology, School of Life Sciences, Sun Yat-sen University, Guangzhou, China; Natural Resources Canada, Canada

## Abstract

The adoption of pest-resistant transgenic plants to reduce yield loss and pesticide utilization has been successful in the past three decades. Recently, transgenic plant expressing double-stranded RNA (dsRNA) targeting pest genes emerges as a promising strategy for improving pest resistance in crops. The steroid hormone, 20-hydroxyecdysone (20E), predominately controls insect molting via its nuclear receptor complex, EcR-USP. Here we report that pest resistance is improved in transgenic tobacco plants expressing dsRNA of *EcR* from the cotton bollworm, *Helicoverpa armigera*, a serious lepidopteran pest for a variety of crops. When *H. armigera* larvae were fed with the whole transgenic tobacco plants expressing *EcR* dsRNA, resistance to *H. armigera* was significantly improved in transgenic plants. Meanwhile, when *H. armigera* larvae were fed with leaves of transgenic tobacco plants expressing *EcR* dsRNA, its *EcR* mRNA level was dramatically decreased causing molting defects and larval lethality. In addition, the transgenic tobacco plants expressing *H. armigera EcR* dsRNA were also resistant to another lepidopteran pest, the beet armyworm, *Spodoptera exigua*, due to the high similarity in the nucleotide sequences of their *EcR* genes. This study provides additional evidence that transgenic plant expressing dsRNA targeting insect-associated genes is able to improve pest resistance.

## Introduction

Plants defend attacks from insect herbivores and, in turn, insect pests damage host plants. In the past three decades, transgenic technology has been developed to generate insect-resistant plants for reducing both yield loss and pesticide utilization [1]. Transgenic plants are becoming vital components of integrated pest management worldwide [2]. There is no doubt that the best example is transgenic plants expressing *Bacillus thuringiensis* (Bt) toxins (Bt plants), which have achieved significant success economically and ecologically. It was reported that transgenic cotton expressing Bt toxin (Bt cotton) greatly suppressed the cotton bollworm, *Helicoverpa armigera*, a destructive pest for cotton and many other crops [3]. Bt toxins kill major target pests and cause little or no harms to vertebrates and most other organisms. However, the sustainability and durability of pest resistance in Bt plants appear to be more and more problematic. With intensive cultivation of Bt crops, increasing pest resistance to transgenic plants were frequently reported [4], [5], for example, increasing *H. armigera* resistance to Bt cotton [6]. Even in transgenic plants expressing two different types of Bt toxins, cross-resistance has been discovered [7]. Moreover, less pesticide utilization after planting Bt cotton lead to outbreak of non-target pests of Bt toxins, such as the mirid bug, *Lygocoris lucorμm* [8]. Although scientists are trying to develop second- and third-generation insect-resistant transgenic plants, no success like Bt plants has been realized yet [1], [2].

In the backdrop of increased insect resistance to Bt plants, it is urgent to develop new techniques for integrated pest management. An attractive approach for crop protection is to use the RNA interference (RNAi) technique to knock down essential genes of insect pests [9]–[12]. Since the discovery that ingested double-stranded RNA (dsRNA) could trigger RNAi in *Caenorhabditis elegans* [13], the molecular mechanism of RNAi has been extensively studied from yeast to insects to mammals, showing functional and evolutionary conservation [14]–[16]. RNAi is now an invaluable tool for reverse genetics study in various organisms, including several orders of insects [12]. For insect functional genomics studies, exogenous dsRNA needs to be delivered to bodies for silencing expression of target genes. In most insects, genes can be knocked down by dsRNA injection [12], [16], although more variability and difficulty were reported in Lepidoptera [17]. Apparently, dsRNA injection is not applicable for pest control in the field, simpler and more convenient means of dsRNA delivery is necessary. It was reported that oral feeding with dsRNA causes RNAi effects in several insects [18], [19]. In a previous study, we showed that oral feeding with bacterially expressed dsRNA of a non-midgut gene, *CHSA*, in the beet armyworm, *Spodoptera exigua*, results in lethality [20]. Recently, it was reported that spraying with dsRNA on the Asian corn borer, *Ostrinia furnalalis*, causes RNAi effects [21]. In plants, a robust RNAi pathway is essential for normal development [22]. Significantly, plants could be armed with dsRNA to fend off insect pests, as transgenic plants producing dsRNAs targeting selected insect genes exhibited suppressive effects on gene expression and caused lethality in *H. armigera* [23], [24], the western corn rootworm, *Diabrotica virgifera* [25], and the pea aphid, *Acyrthosiphon pisum* [26]. Because the introduced dsRNA in transgenic plants can be highly specific to target insects, this approach limits the adverse effects on non-target organisms and exhibits potential application in pest control and crop protection [10], [24].

The steroid hormone, 20-hydroxyecdysone (20E), predominately controls insect molting and metamorphosis via its nuclear receptor complex, ecdysone receptor (EcR) and ultraspiracle (USP) [27]. The ligand-receptor complex, 20E-EcR-USP, triggers a transcriptional cascade, including transcription of the 20E primary-response genes (i.e. transcription factor genes *Br-C*, *E74*, *E75*, and *E93*) and the subsequent 20E secondary-response genes, resulting in molting and metamorphosis [27], [28]. In the fruitfly, *Drosophila melanogaster*, the *EcR* null allele is embryonic lethal [29], and the *USP* hypomorphic allele dies during the embryonic stage or the first larval instar [30]. RNAi knockdown of *EcR-USP* causes significant molting defects and lethality in several insect species [31]–[34]. Because EcR-USP is absolutely required for insect growth and development, we suppose that transgenic plants expressing dsRNA of *EcR* or *USP* might be effective to improve pest resistance.

So far, in the reported transgenic plants expressing dsRNA to improve pest resistance, none of the targeted pest genes is insect-associated. In this study, we discovered that pest resistance is improved in transgenic tobacco plants expressing dsRNA of an insect-associated gene *EcR* from *H. armigera* (*HaEcR*), supporting the idea that transgenic plant expressing dsRNA targeting insect-associated genes is able to improve pest resistance.

## Results

### Ingestion of bacterially expressed *HaEcR* dsRNA results in molting defects and lethality in *H. armigera* larvae

Previously, we showed that oral feeding with bacterially expressed dsRNA in *S. exigua* is able to cause RNAi effects [20]. We also found that, in *B. mori*, injection of *EcR* and *USP* dsRNAs result in significant lethality during the larval-pupal metamorphosis [33], [34]. Thus, we first tested whether oral feeding with bacterially expressed *HaEcR* and *HaUSP* dsRNAs were able to cause molting defects and lethality in *H. armigera* larvae.

**Figure 1:**
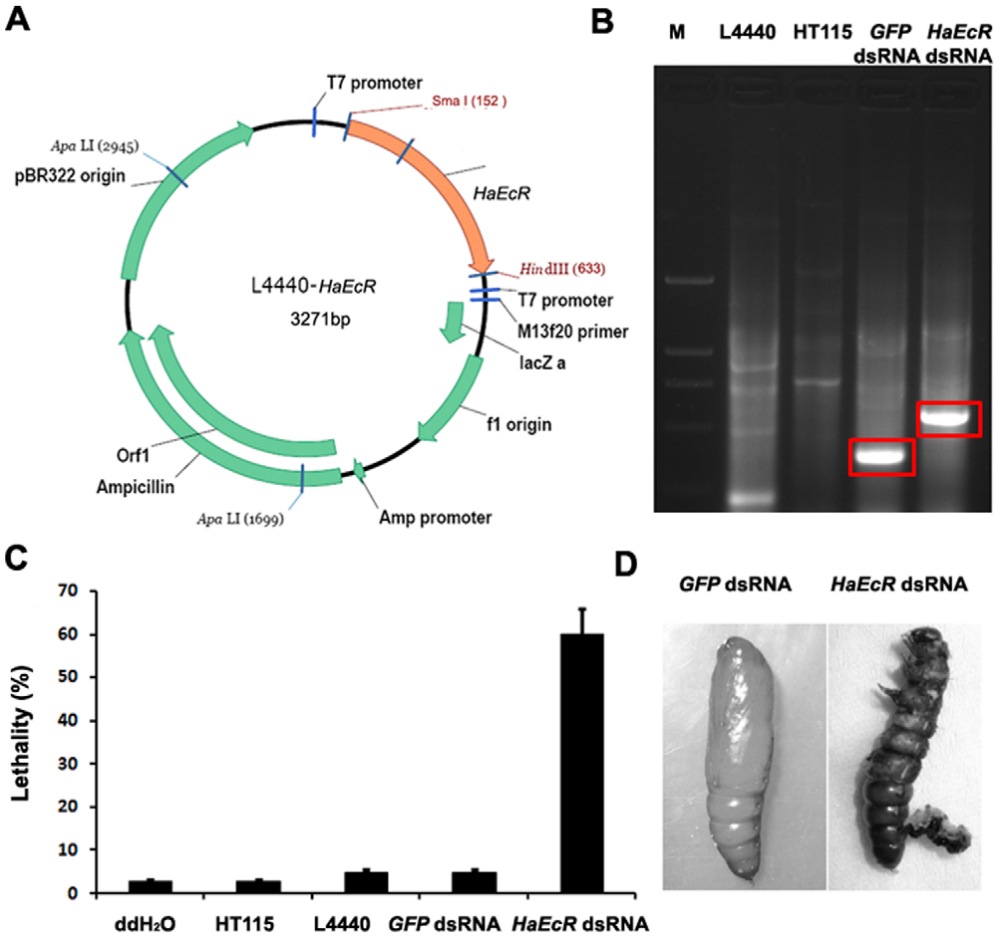
Ingestion of bacterial-expressed *HaEcR* dsRNA results in molting defects and lethality in *H. armigera* larvae. (A) The L4440*-HaEcR* construct producing *HaEcR* dsRNA in *E. coli* HT115. (B) Expression of ***HaEcR*** dsRNA was confirmed by electrophoresis on 1% agarose gel. (C) Ingestion of bacterial-expressed *HaEcR* dsRNA caused up to 60% larval lethality in *H. armigera*. (D) Some *H. armigera* larvae died as larval-pupal intermediates.

Apparently, ddH_2_O and the host bacteria HT115 caused no effects on larval growth and development in *H. armigera*. Likewise, the control dsRNA prepared from HT115 containing the empty vector L4440 and L4440-*GFP* caused less than 5% larval lethality. However, *HaEcR* dsRNA prepared from HT115 containing L4440*-HaEcR* resulted in up to 60% larval lethality (Figure 1C). Most larvae that fed on *HaEcR* dsRNA failed to shed cuticles during larval molting and died with small sizes, or formed larval-pupal intermediates (Figure 1D) with lethal phenotypes similar to *EcR* RNAi in *B. mori* [33]. However, *HaUSP* dsRNA prepared from HT115 containing L4440*-HaUSP* only caused ∼10% lethality, likely due to a comparatively low efficiency of RNAi knockdown.

**Figure 2:**
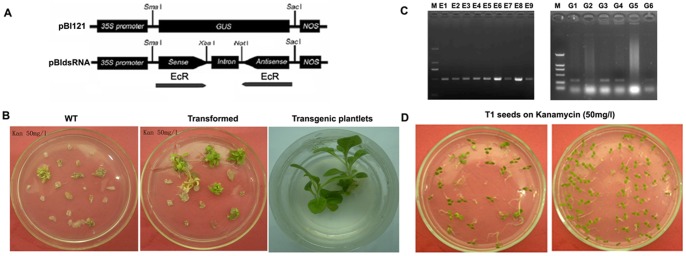
Generation of transgenic tobacco plants producing *HaEcR* dsRNA and *GFP* dsRNA. (A) pBI121-*dsEcR*: the constructed pBI121 vector expressing hairpin *HaEcR* dsRNA in transgenic tobacco plants. See details in materials and methods. (B) Transgenic tobacco plants expressing dsRNAs were obtained using the standard procedure. (C)Independently derived transgenic lines (E1–E9 for *HaEcR* dsRNA; G1–G6 for *GFP* dsRNA) were analyzed by PCR amplifications of the genomic DNA. (D) Homologous transgenic tobacco plants were selected by kanamycin after three progenies. See details in materials and methods.

### Transgenic tobacco plants expressing hairpin dsRNAs grow normally

Since bacterially expressed *HaEcR* dsRNA causes more significant molting defects in *H. armigera* than bacterially expressed *HaUSP* dsRNA, in the following transgenic plants, only the hairpain *HaEcR* dsRNA was expressed. The hairpin *GFP* dsRNA was expressed as a control. The GUS reporter gene in the expression vector, PBI121, was replaced by hairpin dsRNA of either *HaEcR* or *GFP* (Fig 2A), and transgenic tobacco plants were obtained (Figure 2B). Independently derived transgenic lines (E1–E9 for *HaEcR* dsRNA; G1–G6 for *GFP* dsRNA) were analyzed by PCR amplifications, showing that the hairpin dsRNAs were inserted into tobacco genomic DNA successfully (Fig 2C). Homologous transgenic plants were selected (Fig 2D) and used for further experiments. The growth of the transgenic tobacco plants expressing hairpin dsRNA was indistinguishable from that of the wild-type plants.

**Figure 3:**
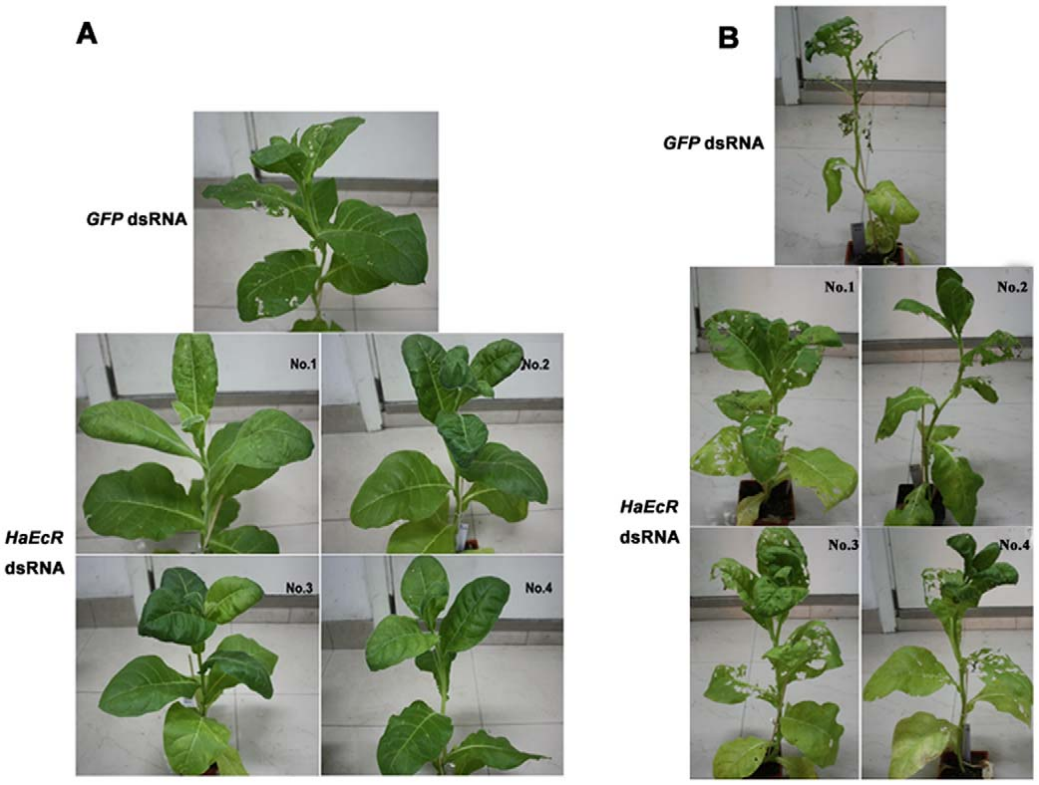
Resistance to *H. armigera* is improved in transgenic tobacco plants expressing *HaEcR* dsRNA. One transgenic tobacco plant expressing *GFP* dsRNA was used as a control, and 4 different transgenic tobacco plants expressing *HaEcR* dsRNA were used as experimental groups. Similar sizes of ∼45-day-old homozygous transgenic plants and day 1 of 2^nd^ instar larvae were utilized in the bioassay. Thirty *H. armigera* larvae were randomly released on the top mature leaves to evaluate dsRNA effects of the whole transgenic plants. After 1 week of feeding, transgenic tobacco plants expressing *HaEcR* dsRNA exhibited higher resistance to *H. armigera* than the control (A). After 3 weeks of feeding, the transgenic tobacco plants expressing *HaEcR* dsRNA are much less damaged (B).

**Figure 4:**
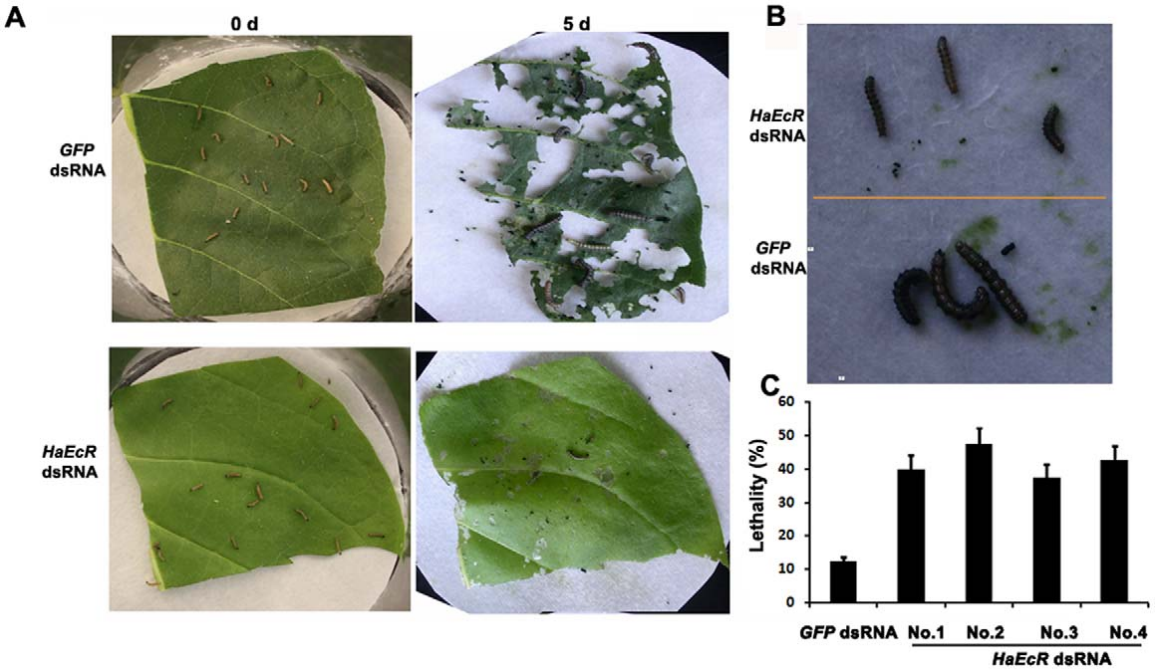
*H. armigera* larvae feeding with transgenic tobacco plants expressing *HaEcR* dsRNA die with significant molting defects. Fifty *H. armigera* larvae were fed with a detached mature leaf maintained in an 80 mm sterile plastic flask. Three similar leaves from the same plant were repeated in a feeding bioassay. The other conditions are the same as Figure 3. (A) Leaves from transgenic tobacco plants expressing *HaEcR* dsRNA were ingested significantly less by *H. armigera* larvae after 5 days of incubation in comparison with those expressing *GFP* dsRNA. (B) The growth of *H. armigera* larvae feeding with transgenic tobacco leaves expressing *HaEcR* dsRNA was significantly delayed and their body sizes reduced. (C) Feeding with transgenic tobacco leaves expressing *HaEcR* dsRNA (No. 1–4) caused significantly higher lethality than in the control.

### Resistance to *H. armigera* is improved in transgenic tobacco plants expressing *HaEcR* dsRNA

To evaluate whether resistance to *H. armigera* was improved in transgenic tobacco plants expressing *HaEcR* dsRNA in comparison with those expressing *GFP* dsRNA, *H. armigera* larvae were randomly released on top mature leaves to feed on the whole plants for weeks. After 1 week of feeding, it was evident that transgenic tobacco plants expressing *HaEcR* dsRNA exhibited much higher resistance to *H. armigera* than the control (Figure 3A). After 3 weeks of feeding, the transgenic tobacco plants expressing *HaEcR* dsRNA are much less damaged (Figure 3B). The insect-feeding trials clearly demonstrated that resistance to *H. armigera* is improved in transgenic tobacco plants expressing *HaEcR* dsRNA.

**Figure 5:**
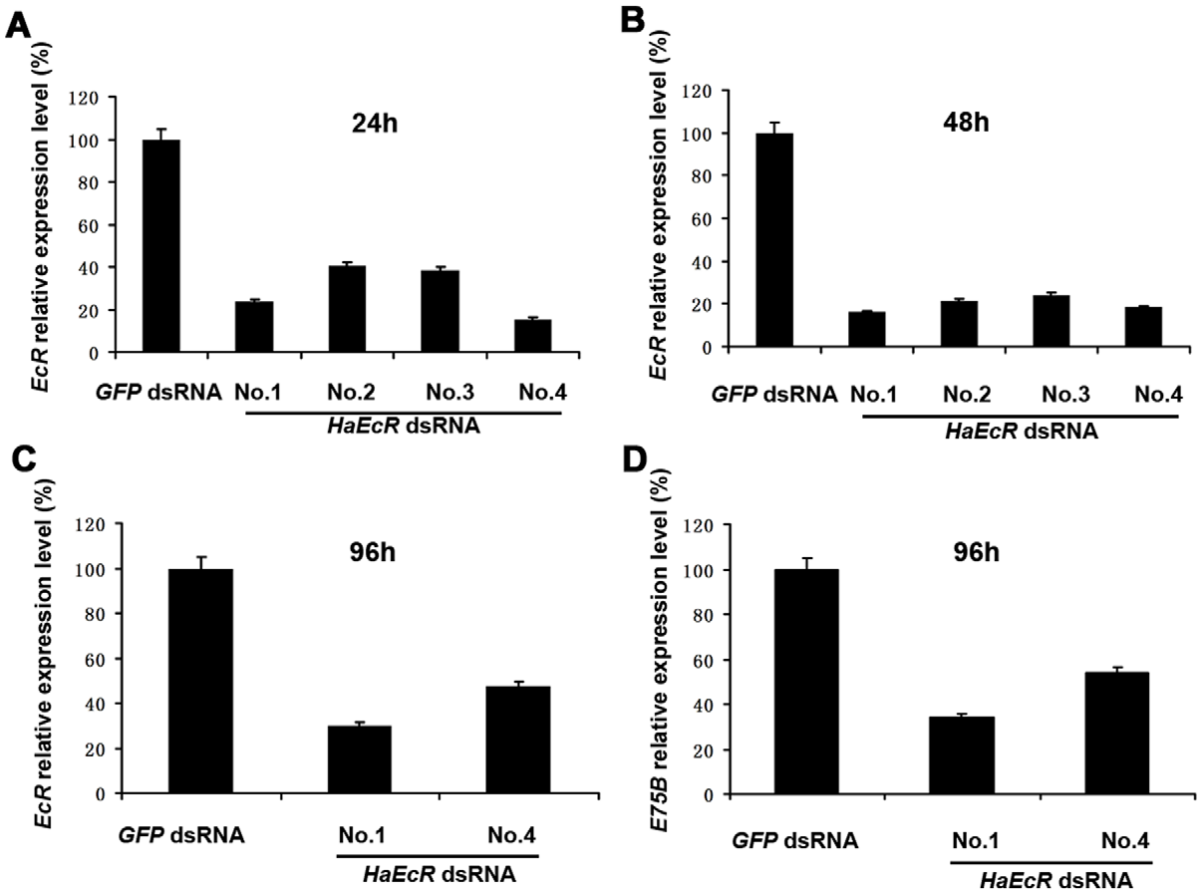
*HaEcR* expression in *H. armigera* is suppressed by feeding with leaves of transgenic tobacco plants expressing *HaEcR* dsRNA.

### *H. armigera* larvae fed with leaves of transgenic tobacco plants expressing *HaEcR* dsRNA die with significant molting defects

Meanwhile, insect-feeding trials with detached mature leaves showed that transgenic tobacco plants expressing *HaEcR* dsRNA were ingested significantly less by *H. armigera* larvae after 5 days of incubation in comparison with those expressing *GFP* dsRNA (Fig 4A). Importantly, the growth of *H. armigera* larvae feeding with leaves of transgenic tobacco plants expressing *HaEcR* dsRNA was significantly delayed and their body sizes reduced, mostly because it took them a much longer time to molt than the control animals (Figure 4B). Moreover, feeding with leaves of transgenic tobacco plants expressing *HaEcR* dsRNA caused significantly higher lethality (40%) than in the control (10%) (Figure 4C). In conclusion, the *H. armigera* larvae feeding with leaves of transgenic tobacco plants expressing *HaEcR* dsRNA died with significant molting defects similar to those feeding with bacterially expressed *HaEcR* dsRNA.

**Figure 6:**
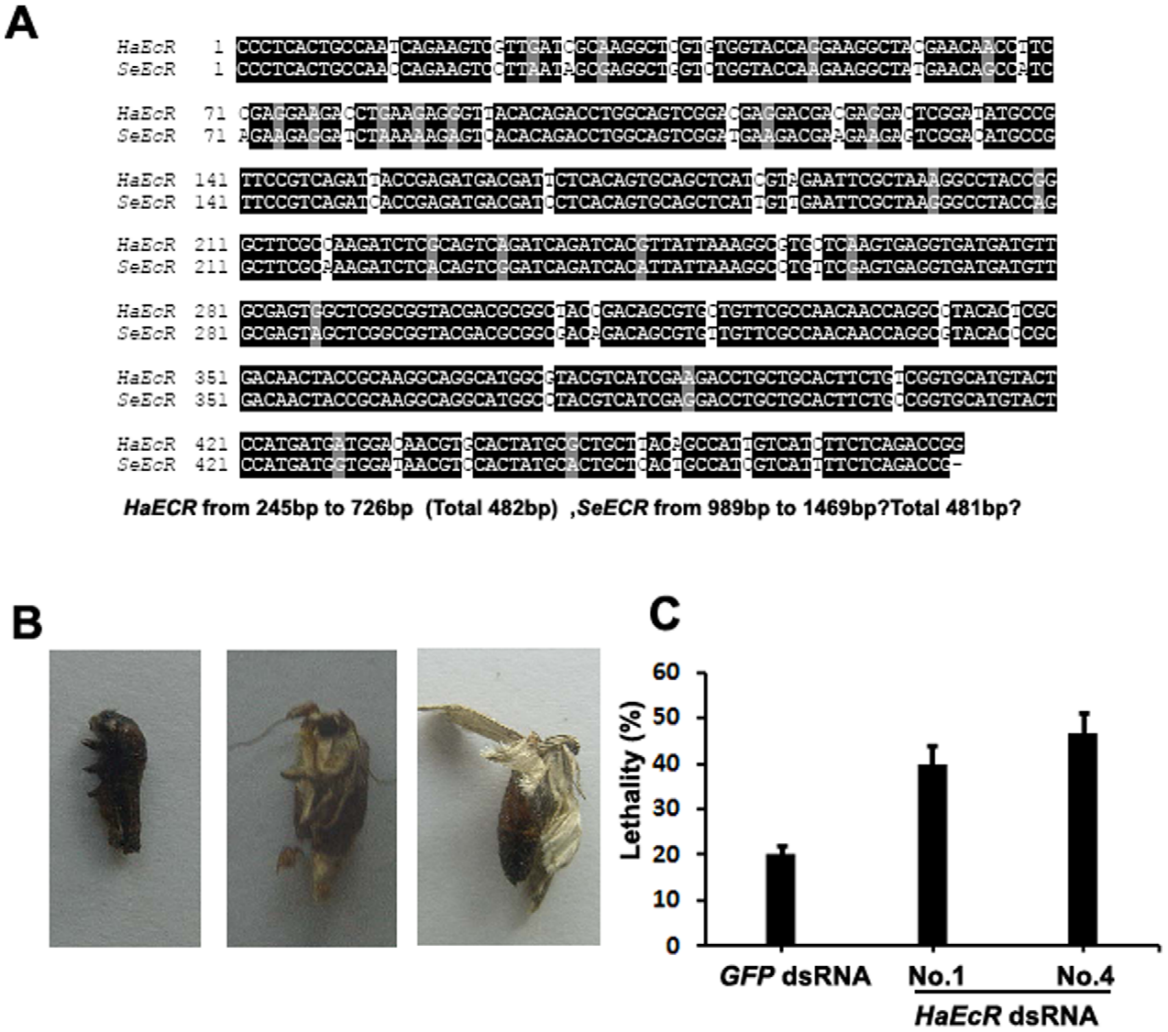
Transgenic tobacco plants expressing *HaEcR* dsRNA improves resistance to *S. exigua*. (A) The nucleotide sequence of *HaEcR* and *SeEcR* share high identity. (B) The *S. exigua* larvae feeding with leaves of transgenic tobacco plants expressing *HaEcR* dsRNA died at different developmental stages with significant molting defects. (C) Feeding with leaves of transgenic tobacco plants expressing *HaEcR* dsRNA caused significantly higher lethality than in control.

### *HaEcR* and *HaE75B* mRNA levels in *H. armigera* are suppressed by feeding with transgenic tobacco plants expressing *HaEcR* dsRNA

We assume that the molting defects in *H. armigera* larvae resulted from suppression of its *HaEcR* mRNA level by feeding with leaves of transgenic tobacco plants expressing *HaEcR* dsRNA. To verify this hypothesis, *HaEcR* mRNA levels in *H. armigera* larvae were measured by quantitative real-time PCR (qRT–PCR). Importantly, *HaEcR* mRNA level significantly decreased in comparison with the control after 24 and 48 hours of feeding, respectively (Figure 5A and B). However, the 20E primary-response gene, *HaE75B* [35], was only slightly downregulated by 48 hours (data not shown), so we extended the feeding assay for 96 hours. By then, both mRNA levels of *HaEcR* (Figure 5C) and *HaE75B* (Figure 5D) were significantly decreased, confirming that the expression of *HaE75B* requires that of *HaEcR* [35]. Taken together, we conclude that suppression of *HaEcR* expression in *H. armigera* by feeding with transgenic tobacco plants expressing *HaEcR* dsRNA interrupts the 20E-triggered transcriptional cascade, results in molting defects, and causes larval lethality.

### Transgenic tobacco plants expressing *HaEcR* dsRNA improves resistance to *S. exigua*

Homology search reveals that the nucleotide sequence of *S. exigua EcR* (*SeEcR*) shares 89% identity to that of *HaEcR* (Figure 6A), suggesting that transgenic tobacco plants expressing *HaEcR* dsRNA might exhibit resistance to *S. exigua* as well. As expected, resistance to *S. exigua* was improved in transgenic tobacco plants expressing *HaEcR* dsRNA (data not shown). The *S. exigua* larvae feeding with leaves of transgenic tobacco plants expressing *HaEcR* dsRNA died during larval molting, pupation, and adult emergence (Figure 6B) showing significant molting defects. Moreover, feeding with leaves of transgenic tobacco plants expressing *HaEcR* dsRNA caused significantly higher lethality (40%) than in the control (20%) (Figure 6C).

**Table 1:** Primers used in this study.

Gene name	Forward primers	Reverse primers
**Real-time**		
*HaActin*	AAGTTGCTGCGCTGGTAGTA	TCTCCATATCGTCCCAGTTG
*HaEcR*	CACTGCCAATCAGAAGTCGT	GGCCTTTAGCGAATTCTACG
*HaE75B*	AGCTCACAACGGACTCACTG	TCTAGCACGCGTTTGAGC
**Gene cloning and vector construction**		
*GFP* **-P1**	CCC**CCCGGG**CGATTTCAAGGAGGACGG	CCC**AAGCTT**CCATGCCATGTGTAATCCC
*GFP* **-P2**	CCC**CCCGGG**CGATTTCAAGGAGGACGG	CCC**TCTAGA**CCATGCCATGTGTAATCCC
*GFP* **-P3**	CCC**GAGCTC**CGATTTCAAGGAGGACGG	CCC**GCGGCCGC**CCATGCCATGTGTAATCCC
*HaEcR* ***-*** **P1**	CCC**CCCGGG**CCCTCACTGCCAATCAGAAGTCGTT	CCC**AAGCTT**CCGGTCTGAGAAGATGACAATGGCT
*HaEcR* ***-*** **P2**	CCC**CCCGGG**CCCTCACTGCCAATCAGAAGTCGTT	CCC**TCTAGA**CCGGTCTGAGAAGATGACAATGGCT
*HaEcR* ***-*** **P3**	CCC**GAGCTC**CCCTCACTGCCAATCAGAAGTCGTT	CCC**GCGGCCGC**CCGGTCTGAGAAGATGACAATGGCT
*HaUSP* ***-*** **P1**	CCC**CCCGGG**GTTCAAGAGGAGAGGCAAAG-3	CCC**AAGCTT**GCCTGTTTTTCAGTCCCTTC
*RTM1* **-P**	CCC**TCTAGA**ACGTTGTAAGTCTGATTTTTGACTCTTC	CCC**GCGGCCGC**TCTATCTGCTGGGTCCAAATCACATATTA

## Discussion

As introduced above, transgenic plants expressing dsRNA emerges as a promising approach for pest control [9]–[12]. In this study, we demonstrated that pest resistance is able to be improved in transgenic tobacco plants expressing dsRNA of the insect-associated gene *EcR*. The ligand-receptor complex 20E-EcR-USP triggers a variety of developmental and physiological events in insects, such as molting and metamorphosis [27], [28]. Because the steroid hormone 20E and its the nuclear receptor complex EcR-USP are insect-associated and absolutely required for insect development, our experimental data suggest that transgenic plants expressing *EcR* dsRNA might effective to improve pest resistance.

We also found that transgenic tobacco plants expressing dsRNA targeting *HaEcR* improved resistance to another lepidopteran pest, *S. exigua*, due to the high identity of nucleotide sequences between *HaEcR* and *SeEcR*. This result implies that a transgenic plant expressing *HaEcR* might improve resistance to *H. armigera*, *S. exigua*, and likely other lepidopteran pests. On the other hand, however, it might be risky to affect non-pest insects, including honeybees and wasps. For biosafety, even in an insect-associated gene such as *EcR*, the 5′– and 3′-untranslated regions, which usually have more variability among different insect species, might be better choices to make hairpin dsRNA than the well-conserved regions chosen in this study [36].

For a long time, EcR-USP was believed to be specific to insects and arthropods in general [37]. However, recently, insect *EcR* homologues have been identified in certain nematodes [37]–[40], which also have molting processes like insects. Since nematodes also cause damage to plants, *EcR*might be a good target gene to control pest nematodes using transgenic plants expressing dsRNA. However, biosafety might be an even more serious issue when nematode genes are possibly targeted by the same dsRNA. Although no *EcR* homologues has been identified in genomes of higher organisms [37], so we assume that, even in an insect-associated gene such as *EcR*, designing hairpin dsRNA for gene targeting must be carefully considered for pest control.

It is necessary to note that we first used bacterially expressed dsRNA to select candidate targeting pest genes. By this means, we found that *HaEcR* is a better candidate RNAi targeting gene than *HaUSP*, because *HaUSP* was relatively difficult to be suppressed by feeding bacterially expressed dsRNA. In the following studies, only *HaEcR* dsRNA was expressed in transgenic tobacco plants. Particular for large-scale screens for targeting pest genes, the bacterially expressed dsRNA system is very useful considering that it is less expensive and time-consuming than transgenic plants.

In summary, in this study we demonstrated that transgenic plants expressing dsRNA targeting insect-associated genes could improve pest resistance, although biosafety is still the most serious issue which needs to be carefully considered.

## Materials and Methods

### Plant and insect culture

Tobacco plants (*Nicotiana tabacum*) were grown in greenhouse at 26±1°C and 60–80% relative humidity under a photoperiod of 16-h-light/8-h-dark. *H. armigera* eggs were obtained from Nanjing Agricultural University. *H. armigera* larvae were reared in controlled chambers at 26±1°C and 75±5% relative humidity under a photoperiod of 14-h-light/10-h-dark using an artificial diet [24]. *H. armigera* larvae were reared as groups prior to the 3^rd^ instar and fed individually since day 1 of 3^rd^ instar. *S. exigua* larvae were reared as previously described in detail [20].

### *HaEcR* dsRNA preparation in *E. coli*

The procedure of dsRNA preparation in *E. coli* was according to the reported studies [20], [41], [42]. To construct a plasmid expressing dsRNA of *HaEcR* (GenBank accession No. EU526831) in *E. coli*, a 482-bp fragment (245–726) was amplified by PCR using *H. armigera* cDNA as a template. The PCR primers contained *Sma* I and *Hin* dIII sites. The *HaEcR Sma* I-*Hin* dIII PCR product was then cloned into the plasmid L4440 [41]. The L4440*-HaEcR* construct (Figure 1A) was transformed into *E. coli* HT115 (DE3) competent cells and cultured overnight in LB medium at 37°C with 100 μg/ml ampicillin and 12.5 μg/ml tetracycline. The culture was diluted 100-fold in 100 ml of 2×YT medium and allowed to grow to OD_595_ 0.4. T7 polymerase was induced with 0.4 mM IPTG and incubated with shaking for additional 4 h at 37°C. The expressed dsRNA was extracted and confirmed by electrophoresis on 1% agarose gel (Figure 1B). For large-scale dsRNA preparations for feeding bioassays of *H. armigera*, 100 ml IPTG-induced culture was concentrated by centrifugation at 10000 g for 2 minutes and the bacteria was resuspended in 800 μl sterile water. The empty vector L4440 and L4440-*GFP* were expressed for preparing control dsRNA in the feeding bioassay. In addition, L4440-*HaUSP* was also expressed for preparing *HaUSP* dsRNA. The primers used in this study are listed in Table 1.

### Transgenic tobacco plants expressing hairpin *HaEcR* dsRNA

To construct a plasmid to express *HaEcR* dsRNA in tobacco plants, the pBlueSctipt SK(–) was used as an intermediate vector. A 120-nucleotide intron of *Arabidopsis RTM1* gene [43] was cloned into the plasmid SK between the *Xba* I and *Not* I sites. The 482-bp *HaEcR* fragments were PCR amplified with *Sma* I and *Xba* I sites (*EcR*+) as well as *Not* I and *Sac* I sites (*EcR*−), and sequentially inserted into the intron-contained SK vector. The intermediate vector was digested using *Sma* I and *Sac* I and then cloned into the pBI121 expression vector. The constructed pBI121 vector expressing hairpin *HaEcR* dsRNA (pBI121-*dsEcR*) contains a CaMV35S promoter, a sense fragment of *HaEcR* cDNA, a 120-nucleotide intron, an antisense fragment of *HaEcR* cDNA, and a NOS terminator (Fig 2A). *Agrobacterium tumefaciens* strain LBA4404 containing the binary plasmid pBI121-*dsEcR* was used for tobacco transformation. The plant transformation was done following the standard procedure [44], and the transformants were selected using 100 mg/l Kanamycin on MS medium. After one month, rooted plants were transferred into a mixed soil with peat, perlite and vermiculite (1∶1∶1 v/v/v), and grown in greenhouse to set seeds. Genomic DNA was extracted from leaves (0.02 g) of wild-type and transgenic tobacco plants and checked by PCR. Transgenic tobacco plants were grown in greenhouse. After about 2 months, collected seeds (T1 progeny) were germinated on MS medium plus 50 mg/l kanamycin for one week to screen heterlogous or homologous tobacco plants. After three progenies were repeatedly selected by kanamycin, homologous transgenic tobacco plants were obtained and used for further experiments. In addition, transgenic tobacco plants expressing hairpin *GFP* dsRNA were obtained and used as the control in the feeding bioassays.

### Feeding bioassays

To investigate dsRNA effects on *H. armigera*, *HaEcR*, *HaUSP* and control dsRNAs were first prepared using the bacterial expression system. The artificial diet was cut into 20 mm×10 mm×2 mm pieces weighing about 0.9 g. For feeding bioassay, each piece of the diet was covered with 50 μl condensed bacteria (v: v 1∶100) expressing dsRNA or plasmid L4440, or 50 μl ddH_2_O. Larva on day 1 of 3^rd^ instar was selected and reared individually with 1 piece of the artificial diet. Thirty biological repeats were used in each treatment. The diet was replaced daily and the results were recorded accordingly.

The dsRNA effects on *H. armigera* were further investigated using transgenic tobacco plants expressing *HaEcR* dsRNA, with transgenic tobacco plants expressing *GFP* dsRNA as a control. Initial experiments revealed no differences among all the transgenic tobacco plants expressing *GFP* dsRNA. In the following experiments, only one transgenic tobacco plant expressing *GFP* dsRNA was used as a control, and 4 different transgenic tobacco plants expressing *HaEcR* dsRNA were used as experimental groups. In the two feeding bioassays, similar sizes of ∼45-day-old homozygous transgenic tobacco plants and day 1 of 2^nd^ instar larvae were utilized [45]. In some experiments, 30 *H. armigera* larvae were randomly released on the top mature leaves to evaluate dsRNA effects of the whole transgenic tobacco plants and the results were recorded weekly. In other experiments, 15 *H. armigera* or *S. exigua* larvae were fed with a detached mature leaf maintained in an 80 mm sterile plastic flask. Three similar leaves from the same plant were repeated in a feeding bioassay and the results were recorded daily.

### qRT-PCR

Total RNA was extracted using Trizol reagent (Invitrogen) according to the manufacturer's instructions. First-stranded cDNA was made from 3 μg of RNA primed by oligo (dT)_18_ using M-MLV reverse transcriptase (Takara, Japan). qRT-PCR was performed in a 20 μl volume using SYBR Green Master Mix (TOYOBO, Japan) and data were analyzed on the Bio-Rad iQTM5 Real-Time PCR Detection System (Bio-Rad, USA) using the following PCR condition: 95°Cfor 3 min, followed by 40 cycles of 95°C for 15 sec, 60°C for 30 sec, 72°C for 15 sec. All qRT-PCR assays were repeated three times [33].

After feeding with leaves of transgenic tobacco plants expressing dsRNA for an indicated time period, larvae were collected for extracting total RNA for qRT-PCR analysis. Three biological replicates were conducted. Each RNA sample is extracted from 3 larvae mixed. Endogenous *HaActin* was used as the inner control. Primers are listed in Table 1.
